# Spotting plants’ microfilament morphologies and nanostructures

**DOI:** 10.1073/pnas.1901118116

**Published:** 2019-06-13

**Authors:** Ana P. Almeida, João Canejo, Urban Mur, Simon Čopar, Pedro L. Almeida, Slobodan Žumer, Maria Helena Godinho

**Affiliations:** ^a^Centro de Investigação em Materiais/Institute for Nanomodelling, Nanostructures and Nanofabrication, Departamento de Ciência dos Materiais, Faculdade de Ciências e Tecnologia, Universidade Nova de Lisboa, 2829-516 Caparica, Portugal;; ^b^Faculty of Mathematics and Physics, University of Ljubljana, 1000 Ljubljana, Slovenia;; ^c^Área Departamental de Física, Instituto Superior de Engenharia de Lisboa, Instituto Politécnico de Lisboa, 1959-007 Lisboa, Portugal;; ^d^Condensed Matter Physics, Jozef Stefan Institute, 1000 Ljubljana, Slovenia

**Keywords:** nematic liquid crystals, tracheary microfilaments, morphology, mechanical properties

## Abstract

The tracheary system of plant leaves is composed of a cellulose skeleton with diverse hierarchical structures. It is built of polygonally bent helical microfilaments of cellulose-based nanostructures coated by different layers, which provide them high compression resistance, elasticity, and roughness. Their function includes the transport of water and nutrients from the roots to the leaves. Unveiling details about local interactions of tracheary elements with surrounding material, which varies between plants due to adaptation to different environments, is crucial for understanding ascending fluid transport and for tracheary mechanical strength relevant to potential applications. Here we show that plant tracheary microfilaments, collected from *Agapanthus africanus* and *Ornithogalum thyrsoides* leaves, have different surface morphologies, revealed by nematic liquid crystal droplets. This results in diverse interactions among microfilaments and with the environment; the differences translate to diverse mechanical properties of entangled microfilaments and their potential applications. The presented study also introduces routes for accurate characterization of plants’ microfilaments.

## Significance

Microfibers existing in the tracheary systems of plants are crucial for the plants to survive. These microfilaments are curled up, forming left-handed helices that make the contour of tubes responsible for the transport of water and nutrients from the roots to the leaves. The microfilaments present mechanical properties that vary from plant to plant despite having similar polygonal-helical shapes and cellulose skeletons. Here we show that the surface morphology of the microfilaments, sensed by nematic liquid crystal droplets, is at the origin of entanglements, which are responsible for the mechanical behavior of microfilaments. This work introduces routes for the accurate characterization of plants’ microfilaments and to produce bioinspired textiles.

**Fig. 1.**
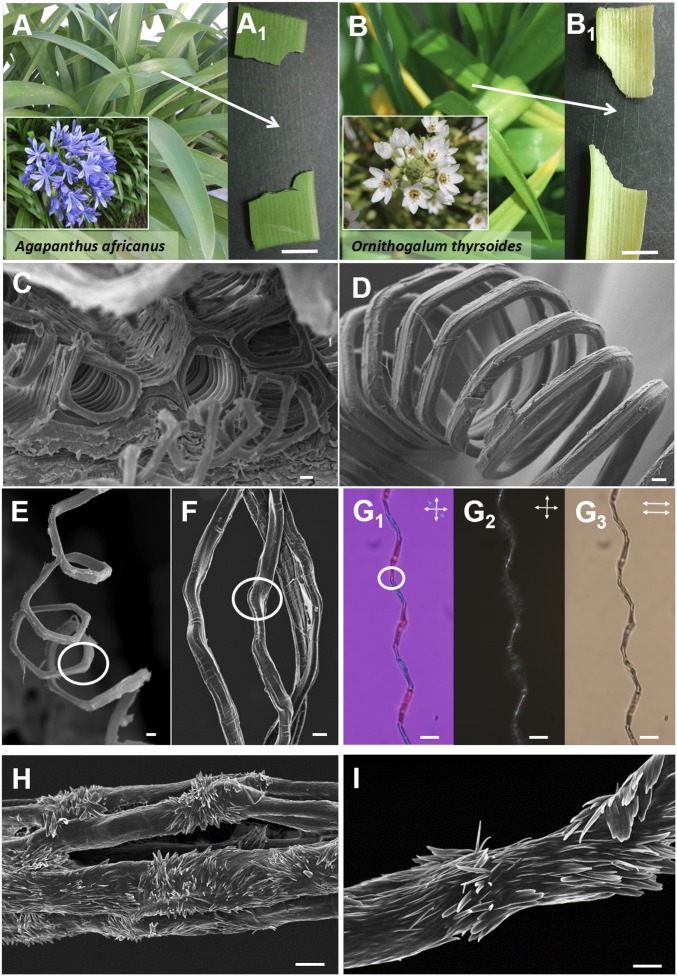
*A. africanus* and *O. thyrsoides* tracheary microfilaments. Photographs of (*A*) *A. africanus* and (*B*) *O. thyrsoides* leaves (main images) and flowers (*Insets*). (*A*_*1*_ and *B*_*1*_) Photographs show bundles of microfilaments drawn from a cut leaf cross section. (*C*) SEM micrograph showing the cross section of an *A. africanus* leaf with helical microfilaments forming a vascular bundle of polygonal tubes. (*D*) SEM picture showing the polygons along a helix collected from an *A. africanus* leaf. (*E* and *F*) SEM images and (*G*_*1*_) POM image taken between cross polarizers with a lambda plate, (*G*_*2*_), cross polarizers, and (*G*_*3*_) parallel polarizers of *A. africanus* stretched microfilaments with elbows highlighted by a white circle. (*H* and *I*) SEM pictures of the *A. africanus* microfilaments after being treated with alkalis. Nanostructures parallel to the main axis of the tracheary filament as well as disordered nanostructures around the elbows are observed. (Scale bars: *A*_*1*_, 0.5 mm; *B*_*1*_, 0.25 mm; *C*, *D*, *F*, and *H*, 2 µm; *E*, 5 µm; *G*_*1*_, *G*_*2*_, and *G*_*3*_, 10 µm; and *I*, 1 µm.)

Microfilaments, which are found in the plant leaves, are a crucial part of plant physiology because they are tightly coiled forming the xylem vessels, tubes through which fluids are transported from the roots to the leaves (1–9). The xylem vessels are present in all plants, and to date, only left-handed helical microfilaments have been isolated (10). It was also reported that the helix diameter depends upon the dimensions of the xylem vessels of the leaves, which is consistent within the species, with individual microfilament diameters of the order of 1.5–2 µm (4). The tubes, made by the microfilaments, appear in tightly packed bundles providing also mechanical strength to the leaves (11). The bundles of helical microfilaments of the tubes are revealed upon breaking the leaves (Fig. 1 *A–C*). Chemical methods or simpler extraction procedures, consisting of breaking the leaves and removing the spiral vessels, are described in literature (4, 6). Due to the abundance of xylem vessels, easy isolation, and low-cost and consistent manufacturing for large-scale mass production, the microfilament helices have been used for biotemplating and for microswimmer fabrication (4, 6, 9). It is worth mentioning that 300 microfilaments are easily isolated from a single *Agapanthus africanus* stem (4).

Plant microfilaments isolated from xylem vessels can have similar diameters to electrospun or spider silk microfilaments. For this reason, similar methods, previously developed for the characterization of microfilaments, found in the animal kingdom or in nonwoven artificial membranes (12–17), can be used to characterize the morphology of microfilaments isolated from the tracheary system of plants. Recently, the responsiveness of liquid crystals to external factors was exploited to sense the surface morphology and the chirality of electrospun cellulose and natural spider silk microfilaments, by application of micronematic droplets (18). Quantitative measurements were also performed with microfilaments that promote planar or helicoidal alignment, inserted in cells with well-defined homeotropic anchoring at the surfaces. Manipulation of the nematic texture with electric and magnetic fields was used to determine the microfilament’s chirality and handedness (18, 19). The correspondence between the nematic liquid crystal anchoring properties of natural microfilaments and their surface morphology and chemical makeup is a challenging open question, as discussed previously in literature (20).

In this work, we establish correlations between mechanical properties and surface morphology of entangled microfilaments, collected from two different plants’ species, *A. africanus* and *Ornithogalum thyrsoides*, which belong to the same *Asparagales* order. Different chemical treatments were imposed to the microfilaments to expose different layers of the surface, before characterizing the surface morphology, by deposition of nematic droplets. This method presents advantages compared with conventional observation techniques such as scanning electron microscopy (SEM), transmission electron microscopy (TEM), and atomic force microscopy (AFM) since it determines surface orientations at the molecular scale without use of sophisticate equipment and is conducted under ambient conditions. It provides insights into small, almost imperceptible surface details of the cellulosic microfilaments. These surface details appear to be crucial for the mechanical behavior of the microfilaments and their interaction with the environment.

## Morphology of Helical Tracheary Microfilaments

For this study, helical tracheary elements extracted from plants belonging to the same *Asparagales* order, *A. africanus* (Fig. 1*A*) and *O. thyrsoides* (Fig. 1*B*), were chosen. The helical tracheary elements were easily isolated and showed very different mechanical properties. Both of them had similar diameters of the order of 1–2.5 µm and were isolated by simply breaking the leaves and pulling the two parts apart (Fig. 1 *A_1_* and *B_1_*). The cross section of the *A. africanus* leaf with vascular bundles, from where the microfilaments were collected, was also observed by SEM (Fig. 1*C*). SEM pictures of single relaxed (Fig. 1*D*) and stretched microfilaments (Fig. 1 *E* and *F*) reveal the polygon-helical shapes that arise due to packing of many such tracheary tubes. The hexagonal packing is the densest packing with the smallest surface/volume ratio. In fact, the corners of the polygonal outline give rise to kinks/elbows when the microfilaments are stretched (highlighted by a white circle in Fig. 1 *E* and *F*).

The microfilaments were also observed stretched under polarized optical microscopy (POM) with cross polarizers and a wave lambda plate, λ = 530 nm (Fig. 1*G_1_*), between cross polarizers (Fig. 1*G_2_*) and between parallel polarizers (Fig. 1*G_3_*). The birefringence of the microfilaments and the blue and orange colors observed, when the sample is seen between crossed polarizers with the lambda plate (Fig. 1*G_1_*), indicate that the cellulosic material, forming the skeleton of the microfilaments, is aligned along their main axis. This was already mentioned in literature for celery tracheary elements (10). However, the black spots observed between crossed and parallel polarizers (Fig. 1 *G_2_* and *G_3_*) along the microfilaments are an indication of a local misalignment, causing light scattering and consequently reduced light transmission. These spots mostly correspond to the elbows, the corners of helical polygons (white circle in Fig. 1*G_1_*), where strain caused by the straightening of the elbow induces local nanostructure disorder in the microfilament. To gather more evidence, the microfilaments were treated with alkalis (*SI Appendix*) to remove the outer layers and expose the inner cellulose skeleton (IR and X-ray diffractograms in *SI Appendix*, Figs. S2 and S3). In Fig. 1 *H* and *I*, we observe cellulose nanostructures aligned with the main axis of the microfilaments as well as clusters of misaligned cellulose nanostructures. Individual cellulose nanostructures (spike-like crystals and platelets of elongated shapes) are observed in the SEM image. These observations are in accordance with the existence of disordered areas induced by stretching of microfilaments from polygonal shaped helices (see also *SI Appendix*, Fig. S1). Similar observations, by AFM, were described in literature but attributed to defects induced when the helical microfilaments with circular cross sections were removed from the leaves (10).

**Fig. 2.**
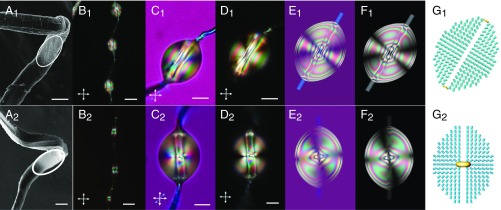
Tracheary microfilament morphologies revealed by nematic liquid crystal droplets. (*A*_*1*_–*G*_*1*_) *A. africanus* and (*A*_*2*_*–G*_*2*_) *O. thyrsoides* microfilaments. *A*_*1*_ and *A*_*2*_ show SEM images of a detail of a stretched filament in which an elbow is seen. The roughness is highlighted by a white circle. The filament from *A. africanus* (*A*_*1*_) presents a rough surface, and the filament from *O. thyrsoides* (*A*_*2*_) presents a smoother one. *B*_*1*_ and *B*_*2*_ show a necklace of nematic droplets suspended along a microfilament, seen by POM under cross polarizers. *C*_*1*_, *C*_*2*_, *D*_*1*_, and *D*_*2*_ show a nematic droplet with homeotropic anchoring at the droplet–air surface under crossed polarizers with a lambda plate and crossed polarizers pierced in *A. africanus* and *O. thyrsoides* tracheary microfilaments. *E*_*1*_, *E*_*2*_, *F*_*1*_, and *F*_*2*_ show numerically simulated transmission micrographs under crossed polarizers with an additional lambda plate and crossed polarizers for droplets pierced in *A. africanus* (*E*_*1*_ and *F*_*1*_) and *O. thyrsoides* (*E*_*2*_ and *F*_*2*_) tracheary microfilaments. Simulated director profile in droplet suspended in an *A. africanus* (*G*_*1*_) and in an *O. thyrsoides* (*G*_*2*_) tracheary filament. Ellipsoidal fringes, typical of homeotropic anchoring on the microfilament surface, can be observed in the nematic droplets pierced in *A. africanus* tracheary microfilaments, while the defect ring around the microfilaments for droplets pierced in *O. thyrsoides* tracheary microfilaments reveals that the microfilament enforces tangential anchoring along the microfilament’s axis. (Scale bars: *A*_*1*_ and *A*_*2*_, 2 µm; *B*_*1*_ and *B*_*2*_, 20 µm; and *C*_*1*_, *C*_*2*_, *D*_*1*_, and *D*_*2*_, 10 µm.)

The untreated microfilaments extracted from leaves of both plants *A. africanus* and *O. thyrsoides* were thoroughly investigated by SEM (Fig. 2 *A_1_–D_1_* and *A_2_–D_2_*, respectively). Both microfilaments exhibit similar diameters, and elbows appear along the helical shapes when stretched. However, the surface morphology of the microfilaments isolated from *A. africanus* (Fig. 2*A_1_*) is distinguished by the presence of nanospherical particles, which gives the filament a rough appearance. The filament isolated from *O. thyrsoides* presents a smoother outer surface (Fig. 2*A_2_*). Nematic droplet chains, dispersed along the microfilaments, were used to spot surface-imposed interactions and morphologies (Fig. 2 *B_1_* and *B_2_*). The droplets were in a size range larger than the anchoring extrapolation length and small enough to be observed individually. The droplets deposited on *A. africanus* microfilaments show ellipsoidal fringes under polarized light (Fig. 2 *B_1_, C_1_*, and *D_1_*), while *O. thyrsoides* microfilaments induce the formation of a defect ring in the middle of the droplet (Fig. 2 *B_2_, C_2_*, and *D_2_*). To extract more quantitative characteristics about the microfilament surface, the polarization microscopy analysis is compared with the simulated optical micrographs based on numerical continuum modeling of nematic structures in droplets with perpendicular molecular orientation at outer interface and pierced by microfilaments with selected anchoring properties (21–23) (*SI Appendix*). With the orientational response of the nematic, the direct signature of the microfilament surface morphologies and interactions (24, 25) was determined. For the microfilaments of *A. africanus,* the typical transmission micrographs with ellipsoidal fringes on both sides of the microfilament imply homeotropic (perpendicular) surface alignment (Fig. 2 *E_1_, F_1_*, and *G_1_*). For surfaces rough on nanoscale, an effective weak perpendicular anchoring occurs independent from the details of molecular interactions. The *O. thyrsoides* microfilaments, which are smooth on nanoscale, impose axial tangential surface anchoring, producing ring defects encircling the microfilaments (Fig. 2 *E_2_, F_2_*, and *G_2_*). The ring is always perpendicular to the microfilaments, even when the droplet is not positioned symmetrically, so that the microfilaments are not piercing through the droplet center. Simulations suggest that the position of the ring reflects the perpendicular anchoring strength at the droplet–air interface; the ring is in the middle of the droplet when the anchoring is strong, but for weaker anchoring, it may be offset toward the droplet edge. This indicates that in our experiments, the anchoring is indeed strong (18). Additionally, the nematic droplets suspended on microfilaments collected from *O. thyrsoides* show a texture similar to droplets pierced on cellulose-based threads (18, 26), while images produced via dispersed nematic droplets on *A. africanus* microfilaments are similar to the images obtained on *Araneidae mangora* microfilaments (18). The droplets are good sensors for the combined effect of surface morphologies and interactions with the microenvironment. The SEM imaging helped to only partially separate the two effects. In addition, the IR spectra of both tracheary microfilaments (*SI Appendix*, Fig. S2) are similar, indicating that they have identical chemical composition and the difference in anchoring is mostly structurally induced.

**Fig. 3.**
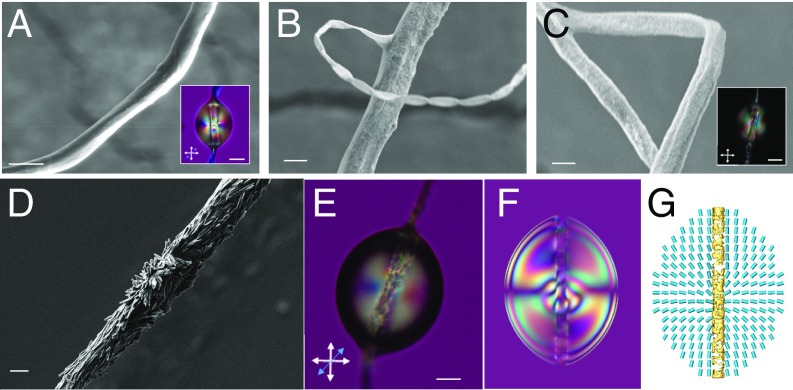
*O. thyrsoides* microfilaments surface nanostructures. (*A*) SEM picture of a tracheary microfilament collected from *O. thyrsoides* leaf with a smooth outer surface. (*B* and *C*) SEM pictures showing microfilaments that were submitted to chemical “peeling” treatment. The *Inset* in *A* represents the same nematic droplet shown in Fig. 2*C_2_*, for comparison with the distinct droplet texture in *C*, *Inset*, seen by POM under cross polarizers, which represents a nematic droplet pierced by a rough microfilament after initial peeling. Notice the development of ellipsoidal fringes, characteristic of homeotropic alignment near the microfilaments. (*D*) SEM picture of a microfilament after further treatment with alkalis. Nanostructures parallel to the main axis of the microfilaments and nanostructures aligned perpendicular to it can be observed. (*E*) POM picture of a nematic droplet pierced in a chemically treated microfilament (*D*) seen between cross polarizers with a lambda plate. (*F*) Numerically simulated transmission micrographs of a droplet pierced by a microfilament with random submicron-scale varying of surface anchoring as seen under crossed polarizers with an additional lambda plate. (*G*) The simulated director profile and decreased nematic order surrounding such a microfilament. (Scale bars: *A*, 5 µm; *A* and *C*, *Insets*, 10 µm; *B*–*D*, 2 µm; and *E*, 20 µm.)

In the next experiments, mechanical removal (sonication) of their outer surface was used, and further alkaline treatment, to expose the internal cellulose structure of the filaments (*SI Appendix*). The smooth outer surface of *O. thyrsoides* microfilaments shown in Figs. 2*A_2_* and 3*A* was mechanically removed, revealing a rougher surface morphology as observed by SEM (Fig. 3 *B* and *C*), which was also detected by the variation of the texture of droplets pierced by the microfilaments (Fig. 3 *A* and *C*, *Insets*). Further alkaline treatment allows the observation of the axially aligned cellulose structures (length 1.1 ± 0.2 µm and diameters of 110 ± 12 nm) with similar size, shapes, and orientation as described for *A. africanus* (Fig. 1 *H* and *I*). As stated before for *A. africanus*, the local misalignment of the nanostructures relative to the main axis of the microfilaments can be attributed to the stretching of the polygon helical shapes. The nanostructures respond to the strain developed at the stretched vertices of the polygonal helices (elbows) where the deformation from the equilibrium radius of curvature is the greatest.

The orientation of the nanostructures on the surface of peeled *O. thyrsoides* microfilaments can also be deduced by observing the texture near the microfilament’s axis in the transmission micrographs. After removing the external layers of the microfilament, dispersed droplets with homeotropic anchoring at the droplet–air surface display a similar texture for both *A. africanus* and *O. thyrsoides* (Fig. 3*E*). This texture is completely different from the textures observed for the previous pierced nematic droplets in “dressed” microfilaments. Experiments show a good qualitative agreement with numerical modeling of droplets with patchy surface orientation varying from patch to patch on submicron scale, as can be observed in Fig. 3 *E–G*.

The pierced large droplets with homeotropic alignment at the liquid crystal–air interface evolve from a ring defect for microfilaments collected from *O. thyrsoides* (Figs. 2*C_2_* and 3 *A*, *Inset*) and ellipsoidal fringes from *A. africanus* (Fig. 2*C_1_*) to a similar rapidly varying texture observed near the microfilaments’ main axis (Fig. 3*E*), which gives evidence of the variation of the nanostructure particle orientation from axial to out of plane and back, also seen by SEM. By depositing droplets of similar sizes at different positions, relatively subtle variations in the microfilament surface morphology are precisely sensed.

The microfilaments of the leaves of both plants have similar cellulosic skeletons surrounded by layers with different morphologies, which were readily distinguished by the nematic droplets. The cellulose skeleton is the main constituent of the microfilaments, and as in composite materials, it determines the mechanical behavior of the helices in the tracheary elements. To establish a relation between structure at the microscale/nanoscale and the macroscopic properties, the mechanical properties of single filaments and bundles of tracheary microfilaments were investigated.

## Mechanical Properties of the Microfilaments

The mechanical properties of fresh leaves stretched along the microfilament’s helical axis direction characterized by uniaxial stress–strain curves were measured, and a brittle behavior was found. Tensile tests were performed for *A. africanus* and *O. thyrsoides* leaves at room temperature (*SI Appendix*, Fig. S4 *A* and *B*). In literature, similar tensile curves were reported for other types of leaves (11, 27). After breaking of leaves, their tracheary elements/xylem vessels are exposed, and their tightly wound microfilament helices start to unwind. The extraction and stretching of microfilament structures lead to the entanglement of nearby helices arising from the same bundle of xylem vessels. In between the two parts of the broken leaf the bundle of xylem vessels is thus transformed in a bundle of entangled microfilaments that are more and more unwounded and stretched with increasing strain. During the leaf stress–strain measurement, the process leads to a nonzero plateau regime (*SI Appendix*, Fig. S4 *A* and *B*), which has a greater magnitude for *A. africanus* than for *O. thyrsoides* leaves.

The microfilaments were carefully isolated, and for assessing the surface mechanical properties of a single microfilament, AFM indentation was used as a less destructive method (28) (experimental details shown in *Materials and Methods*). The elastic modulus of the surface layer of single helical microfilament isolated from *A. africanus* is slightly higher than for *O. thyrsoides*. On the other side the *A. africanus* microfilaments appear to be stiffer (*SI Appendix*, Table S1). The greatest difference was found in adhesion values; *A. africanus* microfilaments presented a much higher value than *O. thyrsoides.* The values obtained indicate that the layers covering the cellulosic skeleton of the microfilaments determine their interaction with the AFM tip. The smooth layer surrounding the microfilaments of *O. thyrsoides* grants greater flexibility and less resistance to indentation, while the outer rough layer of the *A. africanus* microfilaments has a higher affinity to cling to the tip of the AFM probe (Fig. 2 *A_1_* and *A_2_*). Indentation by AFM was also performed to access the mechanical properties of different leaves, and also adhesion was found to vary enormously depending on the material coating (29).

**Fig. 4.**
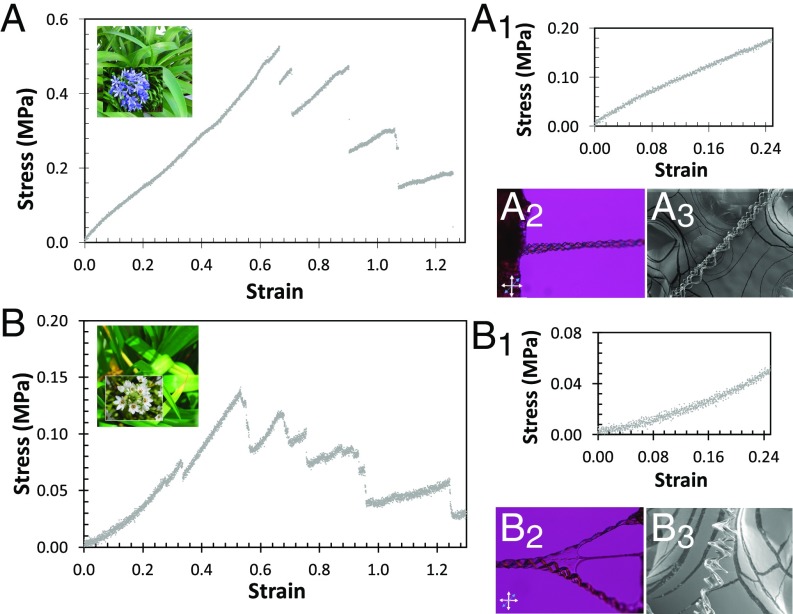
Tensile properties of *A. africanus* and *O. thyrsoides* microfilaments. (*A* and *B*) Typical stress–strain uniaxial curves of *A. africanus* and *O. thyrsoides* bundles of microfilaments, respectively. (*A*_*1*_ and *B*_1_) Zooms of curves in *A* and *B*, respectively, for small values of strain. The strain rate was 0.5 mm/min. Bundles of *A. africanus* and *O. thyrsoides* microfilaments: (*A*_*2*_) *A. africanus* and (*B*_*2*_) *O. thyrsoides* POM pictures between cross polarizers and a lambda plate near the edge of the leaf when the microfilaments were pulled out and (*A*_*3*_) *A. africanus* and (*B*_*3*_) *O. thyrsoides* SEM pictures of suspended, stretched bundles. The black arrows in *A* indicate the maximum stress of two consecutive breaking of bundles. The tensile texts strain rate in *A*, *A*_*1*_, *B*, and *B*_*1*_ was 0.5 mm/min. (Scale bars: *A*_*2*_, *B*_*2*_, and *B*_*3*_, 50 µm, and *A*_*3*_, 100 µm.)

To go beyond probing of surface mechanical properties of microfilaments, the microfilament bundles’ response to uniaxial tensile stress was investigated. The stress/strain curves for uniaxial tensile tests at a strain rate of 0.5 mm/min are shown in Fig. 4. The curves represent typical results for an average of six bundles of *A. africanus* and *O. thyrsoides*, with diameters ranging from 65 to 140 µm supported on a frame similar to that shown in *SI Appendix*, Fig. S5. When the heterogeneous bundles, formed not only by the microfilaments but also by other constituents, such as water and nutrients (sap), are put under tensile load, the bundles break separately. After the break of one bundle the stress suddenly drops. The remaining bundles acquire the tension load, and the stress starts to increase again until the breaking of another bundle, as exemplified by arrows in Fig. 4*A* for *A. africanus*.

The uniaxial tensile curves obtained for bundles extracted from *A. africanus* and *O. thyrsoides* are quite different. The stress–strain curve for *A. africanus* bundles (Fig. 4 *A* and *A_1_*) shows an initial elastic behavior, which allows the estimation of a Young’s modulus of the order of E_bA_ = 0.70 ± 0.05 MPa that is not a well-defined quantity as packing of the microfilaments in the bundle is not tight and also depends on tension. Knowing the number of constituting microfilaments and their cross section we recognize that the active cross section is of about 1,000 times smaller. Therefore, the Young’s modulus of a single microfilament is roughly estimated, being of the order of E_fA_ = 750 ± 50 MPa. We take into account that each bundle (with an initial diameter of the order of 100 µm) is formed, on average by six microfilaments (estimated by SEM photos) with average diameter of 2 µm. After the steep stress/strain linear dependence, a slightly lower slope of linear behavior was observed, reaching its maximum stress at rupture around 0.53 MPa. The maximum jump of all measured steps is a good estimation for the single bundle rupture strength. A small jump means that the remaining nonbroken bundles are still under tension. In this way the rupture strength of a single bundle would be of the order of 0.2 MPa and consequently of ∼200 MPa for microfilaments. In Fig. 4 *A_2_* and *A_3_* a bundle of stretched microfilaments is shown.

Contrasting with the shape of the stress/strain curves observed for the *A. africanus* bundles is the shape of the curves obtained for *O. thyrsoides* bundles (Fig. 4 *B* and *B_1_*). The *O. thyrsoides* tensile tests show that initially small increases in stress give large deformations (Fig. 4*B_1_*). However, at larger strains the material becomes stiffer, and the curve is convex (superlinear). The diverse mechanical behavior observed for the extraction plateau values (*SI Appendix*, Fig. S4*A*) for the stretched bundles of *A. africanus* and *O. thyrsoides* is due to the rough and smooth microfilament nanostructures differences, which were evidenced by AFM and LC droplets. Moreover, the significantly low values of stress for low strains found for *O. thyrsoides* bundles are attributed to the existence of a soft material that was mainly noticed in *O. thyrsoides* bundles (Fig. 4 *B_2_* and *B_3_* and *SI Appendix*, Fig. S4). For small deformations the *O. thyrsoides* bundles behave as a lightly cross-linked rubber until the helical microfilaments get together to form the bundles (Fig. 4 *A_2_* and *A_3_* and *SI Appendix*, Fig. S4*A*). Treating the data according to the same procedure used for *A. africanus*, the Young’s modulus of the bundles and microfilaments for *O. thyrsoides* were estimated from the second part of the linear curve indicated in Fig. 4*B*, E_bO_ = 0.20 ± 0.02 MPa, and for microfilaments, E_fO_ = 125 ± 10 MPa. As observed for *A. africanus* bundles, following these stress/strain linear dependences, others were observed with a decreasing slope. The maximum stress rupture was calculated and is of the order of 0.15 MPa. Once again, the rupture strength of a single bundle is estimated to be of the order of 0.05 MPa and consequently of ∼30 MPa for microfilaments.

The values of the Young’s modulus measured for *O. thyrsoides* bundles are much smaller than the values found for *A. africanus* bundles, which can be partially attributed to the much larger adhesion values found for *A. africanus* microfilaments, by AFM. The skeletons of the microfilaments, for *A. africanus* and *O. thyrsoides*, are very similar, as observed by SEM in Figs. 1*H* and 3*D*, respectively. The differences between the strength of the bundles, from *A. africanus* and *O. thyrsoides*, are mainly due to the surface morphology of the individual microfilaments of each plant. The microfilaments from *O. thyrsoides* are smoother and thus allow their sliding resulting in a lower Young’s modulus of the bundles, compared with the mechanical properties of the bundles collected from *A. africanus* microfilaments, which have a rough surface.

The values for the Young’s modulus of the microfilaments measured by indentation are much smaller (about 1,000 times) compared with the tensile measurements. The same trend was reported in literature for other soft biological systems (30). The difference in the values of the Young’s modulus between the two experimental techniques confirms the nonhomogeneity of the microfilaments. While the stress strain experiments take into account the mechanical properties of the cellulose skeleton and the interaction of the filaments, the AFM results concern the mechanical characteristics of the outer surface of the microfilaments. In fact, the outer surface of the microfilaments, which is soft and sticky, is found crucial for the interactions of the microfilaments bundles and is precisely sensed by the LC droplets and confirmed by the stress–strain experiments.

## Summary

It is known that the surface morphology plays a crucial role on microfilament properties meaning diverse mechanical properties on natural and bioinspired man-made textiles. A simple method of observing nematic liquid crystal droplets threaded by microfilaments, already used for determining their different surface morphologies, was also used in this work for microtracheary elements. The helical threads, with identical diameter, chemical constitution, and internal cellulose skeleton, were collected from two different plants, *A. africanus* and *O. thyrsoides*. SEM images and textures observed for pierced nematic droplets were evidence that the *A. africanus* microfilaments presented a rough outer surface, while the outer surface of the *O. thyrsoides* microfilaments were smooth. Peeling the microfilaments and changing their outer surface morphologies allowed testing the sensitivity of the nematic droplets to their surface morphologies. A similar skeleton formed mainly by cellulose nanostructures was found for both microfilaments. From simple polarization micrographs, supported with numerical modeling imaging, we could associate specific textures of the liquid crystal droplets with different surface morphologies of the microfilaments. The mechanical stress–strain behavior of the leaves and microfilaments was evaluated. While the leaves from *A. africanus* and *O. thyrsoides* presented a brittle similar behavior, their bundle and microfilament mechanical behavior was quite different. The presence of different morphologies on the outer surfaces of the microfilaments was found responsible for the mechanical responses observed. The huge difference found in the *A. africanus* and *O. thyrsoides* bundle mechanical properties are qualitatively understood. The microfilaments sliding, one against the other, which occurs in case of smooth interfaces, for *O. thyrsoides* microfilaments, allows their break one after the other, yielding much lower strength. Particularly relevant is that the skeletons of the microfilaments, in both cases, look practically identical. These reasonable explanations need to be tested in future studies.

The existence of tracheary polygon helices was also made in evidence by POM and SEM images obtained from stretched microfilaments. When a helically winded cellulosic fiber forms a tube (xylem vessels), it would be mechanically the strongest if it would have a circular cross section without sharp bends. Such a tube with a circular cross section would also have the best surface to volume ratio optimal for fluidic transport properties. Nevertheless, packing together several tubes in a solid structure (bundle) providing stability of leaves requires complete filing of the space without a soft material in between. That requires polygonal cross section of tubes, and hexagonal cross section is the closest to the circular one optimal for a single tube.

The main physiological role of the xylem microhelical microfilaments is that they constitute the vessels needed to transport water and mineral nutrients from the roots to the leaves. In this work we gave some insights about the microfilaments’ surface morphologies and their interactions with the environment that open new routes to correlate their structural properties with their mechanical response but not enough to be conclusive about transport properties. The efficiency of the upward transport of the xylem sap should not only be dependent on the internal surface to volume ratio of the vessels but is also affected by the interactions with the surface of the microfilaments. The extent of this influence in different plant species would require a separate dedicated study.

## Materials and Methods

Filaments collected from *A. africanus* and *O. thyrsoides* leaves by simple breaking of the leaves (cross section of the leaves with vessels and filaments for *A. africanus*; Fig. 1 *C* and *D* and *SI Appendix*, Fig. S1 *A*– *C*), similar to the method described in ref. 6, were suspended in 1-cm^2^ frames and treated with NaOH 5% wt/wt solution for 1 h to access the nanostructures cellulose skeletons as shown in Fig. 1 *H* and *I* and *SI Appendix*, Fig. S1 *D*–*F* (skeleton from *A. africanus* filaments) and Fig. 3*D* (skeleton from *O. thyrsoides* microfilaments). The initial *O. thyrsoides* microfilaments, suspended in 1-cm^2^ frames, were also gently treated with water and sonicated during 1 h to remove the smooth outer surface (Fig. 3 *B* and *C*). The leaves from *A. africanus* and *O. thyrsoides* were collected from the garden near Faculty of Science and Technology and gardens in Caparica region, Portugal. All of the microfilaments used in this work were extracted from the leaves on the same day that the leaf was collected from the plant.

The photos of Fig. 1 *A* and *B* and respective insets were taken under solar light with a Canon EOS 450D camera equipped with a 60-mm microlens. The morphology of the microfilaments was observed with a scanning electron microscope CrossBeam Workstation (SEM-FIB)–Zeiss Auriga. The SEM images under the in-lens mode have been carried out with an acceleration voltage of 2 kV and aperture size of 30 μm. A thin carbon layer (<20 nm) was deposited on the suspended microfilaments using a Q150T ES Quorum sputter coater.

The nematic liquid crystal *4′-n-pentyl-4-cyanobiphenyl* (5CB) was used to produce the microdroplet necklaces. The pierced droplets were observed in transmission mode under a polarized optical microscope (POM) Olympus BX51 coupled to Olympus DP73 CCD camera and acquired with the Stream Basic v.1.9 Olympus software. A cold illumination source generated by a halogen lamp (KL 2500 LCD; Olympus) was used. The images were obtained with ×10, ×20, or ×50 objectives (MPlanFL N; Olympus) and automatically scaled by the software. The liquid crystal (LC) necklaces were prepared according to similar protocols reported in ref. 18. A tip of a metallic copper wire, with a 0.1-mm diameter, was used to generate the LC droplets and to deposit them on the suspended microfilaments. The diameter of the droplets was in the range of 5–50 µm.

The mechanical properties of the leaves were examined using uniaxial tensile testing. These tests were carried out using a machine from Rheometric Scientific (Minimat Firmware 3.1) with a 20 N load cell. Tensile tests were performed for *A. africanus* and *O. thyrsoides* leaves at room temperature using a speed of 5 mm/min. For each measurement, five samples collected by cutting the leaves parallel to the trachearies were stretched along the same direction. The samples of *A. africanus* and *O. thyrsoides* used to obtain the nominal stress–strain curves, *SI Appendix*, Fig. S4 *A* and *B*, respectively, had initial lengths of 5.5 and 3.2 mm and cross sections of ∼12.5 × 0.55 mm^2^ and 9.5 × 0.70 mm^2^, respectively.

AFM measurements probing surface mechanical properties of isolated helical microfilaments from bundles of each plant were done with an Asylum Research MFP-3D Standalone using commercial silicon probes (Olympus AC240TS; f_0_ = 70 kHz; k = 1.7 N/m) calibrated with the *Sader* method (31). The isolated microfilaments isolated from each plant were glued onto glass microscopic slide bars using two-sided glue tape.

The acquired AFM curves were analyzed with the Asylum Research tool packages installed in the Igor Pro-6.34 analysis software (Wavemetrics), through which the elastic modulus (ratio between stress and strain) of the surface layer microfilaments and the probe–sample adhesion were determined. A standard force curve was recorded with the AFM indentation measurements, and then the corresponding load versus indentation curve was calculated. To take into account surface–sample heterogeneity, measurements were taken in at least two different regions where 25 force curves were recorded over a regular grid over 5 × 5 μm. Each force curve was taken at a constant vertical displacement speed of 2 μm/s. The maximum applied load was kept constant throughout all measurements, and mechanical properties were extracted from the last 100 nm of indentation through a software trigger that made sure the curve was in the linear force vs. indentation region.

It should be noted that the indentation measurements were not performed in quasi-static conditions, and therefore, the viscous response (loss modulus) might play a significant role; moreover, the calculated absolute values of elasticity are prone to several uncertainties and nonidealities (e.g., in the tip geometry or the surface characteristics of the microfilaments). Nevertheless, all AFM measurements reported in this paper were taken with the same cantilever and the same experimental conditions.

Numerical modeling details and any associated references are available in *SI Appendix*.

## Supplementary Material

Supplementary File
