# Supplementary material for: Spotting plants’ microfilament morphologies and nanostructures
Source: Proc Natl Acad Sci U S A. 2019 Jun 13;116(27):13188–93. doi: 10.1073/pnas.1901118116 (PMC6613095; doi:10.1073/pnas.1901118116)
Supplement: Supplementary File [file pnas.1901118116.sapp.pdf]

Supplemental Information for

Spotting plants microfilaments morphologies and nano structures

Ana P. Almeida[1], João Canejo[1], Urban Mur[2], Simon Čopar[2], Pedro L. Almeida[1,3], Maria Helena Godinho[1*] and Slobodan Žumer[2,4*]

Correspondence to: mhg@fct.unl.pt and slobodan.zumer@fmf.uni-lj.si

This PDF file includes:

Supplemental Text
Figs. S1 to S5
Table S1

www.pnas.org/cgi/doi/10.1073/pnas.1901118116

Characterization of the plants microfilaments

The Infrared (IR) spectra, of the microfilaments as collected from *A. africanus* and *O. thyrsoides* leaves were acquired, Fig. S2 *A,* and after alkali treatment for *A. africanus* microfilaments, Fig. S2 *B.* The analysis was performed using an Attenuated Total Reflectance (ATR) sampling accessory (Smart iTR) equipped with a single bounce diamond crystal on a Thermo Nicolet 6700 Spectrometer. The spectra acquisition was made with a 45° incident angle in the range of 4000 – 525 cm^{-1} and a 4 cm^{-1} resolution. The microfilaments were suspended in a frame of 1 cm^2.

The spectra from *A. africanus* and *O. thyrsoides* microfilaments given in Fig. S2 *A* present peaks at 1735 cm^{-1} and 1247 cm^{-1} characteristic of a carbonyl band and symmetric bridge stretching of uronic ester groups, respectively, which are an indication of the presence of hemicellulose. The peaks characteristic of the absorption band of the aromatic rings from lignin appears around 1510 cm^{-1} and 1595 cm^{-1}. A small band about 1460 cm^{-1} can also be observed for both samples and attributed to $–CH_3$ deformation in lignin and hemicellulose. The weak peak observed at 925 cm^{-1} for *A. africanus* microfilaments could be associated to the adhesion properties detected for *A. africanus* and not for *O. thyrsoides* microfilaments.

In Fig. S2 *B* the decrease of the bands corresponding to lignin and hemicellulose confirms that the chemical treatment promoted the delignification and the hemicellulose extraction of the *A. africanus* microfilaments (1, 2).

Structural characterization of the isolated microfilaments from *A. africanus* leaves, before and after alkali treatment (NaOH 5% wt/wt for 1h and 3h) (Fig. S3), was performed with a X'Pert PRO (PANAlytical) X-ray diffractometer (XRD). The measurements were done using Cu Kα radiation in the range 2θ = 5–50°. The microfilaments were suspended in a square frame of 1 cm^2.

The new peak that appears at ~23º associated to the peak existing at 26º reveals an increase of the crystallinity, which is consistent with the removal of the amorphous part and the presence of the cellulose skeleton formed by nanostructures (3).

Mechanical characterization

To examine the mechanical behaviour of the bundles, obtained by breaking the leaves between two clamps, they were collected in a plastic frame and precisely cut with a cutting machine, in order to fix the initial length l_o=6.5 mm. A homemade tensile apparatus, equipped with a load cell with a precision of 1 cN, was used to obtain tensile load/deformation curves at 0.5 mm/min strain rates. The same experimental procedure was used for the bundles from both plants. The developed force by the imposed deformation was measured for 5 to 6 bundles mounted together in the frame, which were then stretched until rupture. At least five leaves of *A. africanus* and *O. thyrsoides* were used for each tensile essay. The number of bundles, number of microfilaments in each bundle and the cross section of the bundles and of the microfilaments were determined with Image J, version 1.45s, http://imagej.nih.gov/ij/ taking into account the POM and SEM images.

The stress was calculated considering that each bundle has in average 6 microfilaments for *O. thyrsoides* and 6 microfilaments for *A. africanus*, with bundles cross section areas of 133 μm^2 for *A. africanus* and 69 μm^2 for *O. thyrsoides* (at least 10 measurements were made for each bundle and each plant). The average cross section area of the microfilaments was determined for both plants by SEM being the value of 2 μm^2 used for calculations.

Photos and movies of the tensile tests were obtained with a Canon EOS 450D camera equipped with a 60 mm micro-lens.

Numerical modelling

The simulation of the nematic liquid crystal ordering in droplets relies on continuum modelling using tensorial Landau–de Gennes (LdG) free energy minimization approach (4, 5), combining bulk free energy and surface free energy, which accounts for the anchoring on the droplet surface and on the droplet–microfilament interface. The same values of liquid crystal material constants as in reference (6) were used. The free energy was minimized numerically by using an explicit Euler relaxation finite difference scheme. The anchoring strength and type on the microfilament and on the droplet's surface was varied. We used homeotropic, planar and random anchoring at the microfilament surface, and homeotropic on the droplet-air interface. Perlin noise was used to produce randomized, but locally spatially correlated preferred surface orientation. For simulation of the transmission image, transmission polarization micrographs are calculated from the simulated director fields by the improved Jones' method using non-parallel rays (7). Appropriate viewing directions and the angles of polarizer and analyser were selected to match the experiments and the droplets were scaled to match the experimental sizes. Light beam was focused on the centres of the droplets. The values of LC refractive indices used in simulations were set to $n_o = 1.54$ and $n_e = 1.75$. The birefringence of the microfilament was set to $n_o = 1.540$, $n_e = 1.595$ with optical axis pointing along the direction of the microfilament.

References:

1. Oh SY, Yoo DI, Shin Y, & Seo G (2005) FTIR analysis of cellulose treated with sodium hydroxide and carbon dioxide. *Carbohydrate Research* 340(3):417-428.
2. Pan Y*, et al.* (2011) Structural characteristics and physical properties of lotus fibers obtained from Nelumbo nucifera petioles. *Carbohydrate Polymers* 85(1):188-195.
3. Hu TQ, Hashaikeh R, & Berry RM (2014) Isolation of a novel, crystalline cellulose material from the spent liquor of cellulose nanocrystals (CNCs). *Cellulose* 21(5):3217-3229.
4. Gennes PGd & Prost J (1995) *The Physics of Liquid Crystals* (Clarendon Press).
5. Ravnik M & Žumer S (2009) Landau–de Gennes modelling of nematic liquid crystal colloids. *Liq Cryst* 36(10-11):1201-1214.
6. Aguirre LE*, et al.* (2016) Sensing surface morphology of biofibers by decorating spider silk and cellulosic filaments with nematic microdroplets. *Proceedings of the National Academy of Sciences* 113(5):1174-1179.
7. Mur U*, et al.* (2017) Ray optics simulations of polarised microscopy textures in chiral nematic droplets. *Liq Cryst* 44(4):679-687.

Fig. S1

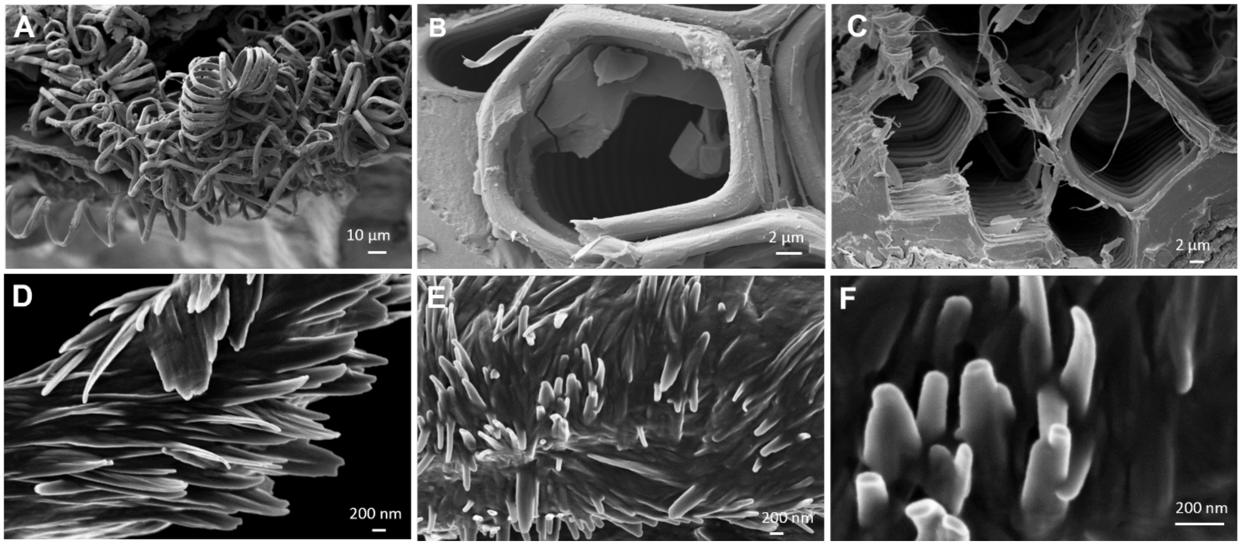

Fig. S1. SEM micrographs of filaments collected from *A. africanus* leaves (*A)* bundle of filaments and (*B* and *C)* cross-section of the leave with filaments and vessels polygonal shapes (in average each polygonal helical microfilament has 5 neighbour helices) (*D* to *F) A. africanus* filaments after being treated with alkalis. Nanostructures parallel to the main axis of the tracheary microfilament as well as nanostructures aligned perpendicular to the main axis of the microfilament can be observed.

Fig. S2

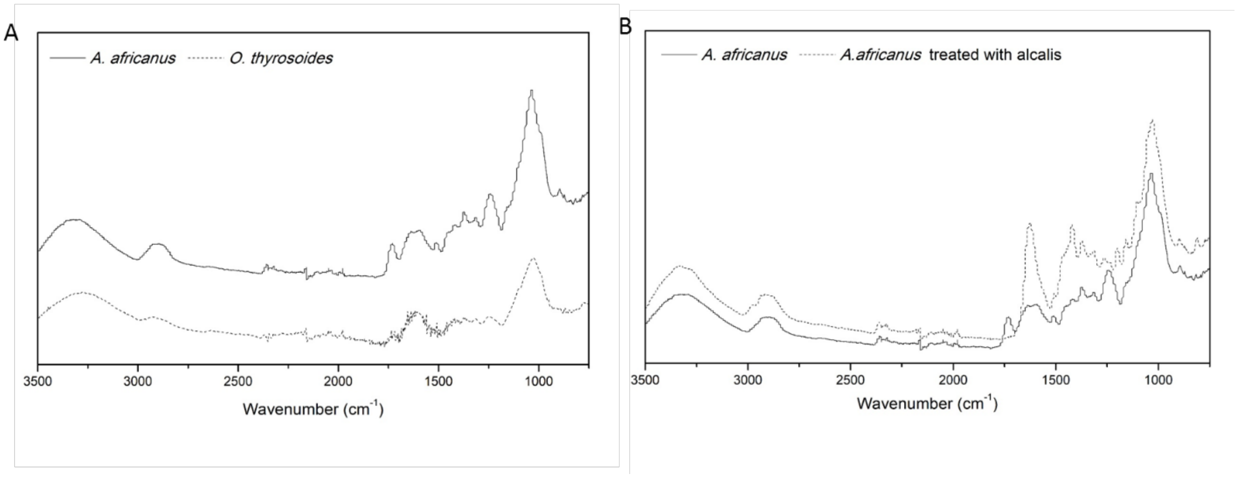

Fig. S2. IR spectra of the microfilaments collected from (*A*) *A. africanus* and *O. thyrsoides* leaves. (*B*) Spectra from *A. africanus* and *A. africanus* microfilaments after treatment with alkalis for 1h.

Fig. S3

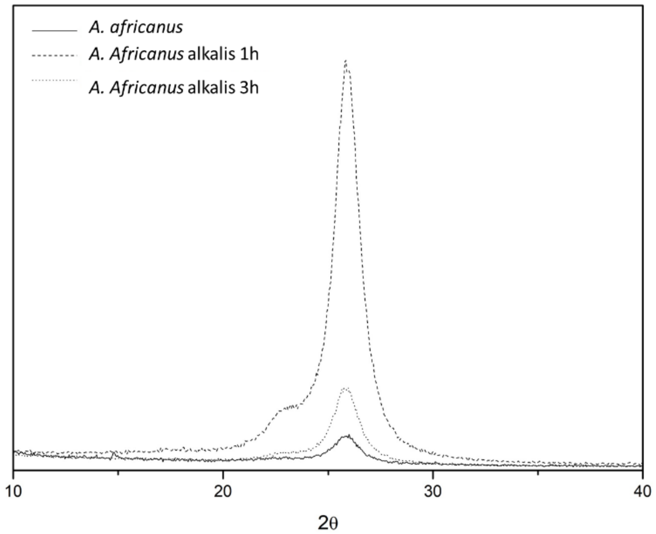

Fig. S3. Xray diffractograms acquired after and before alkalis treatment of the microfilaments isolated from *A. africanus* leaves. The peaks indicate that the alkalis treatment allows the isolation of the cellulose skeleton nanorods confirming the SEM observations (Fig.1 *H* and *I*, Fig.3 *D* and Fig. S1 *D* to *F*).

Fig. S4

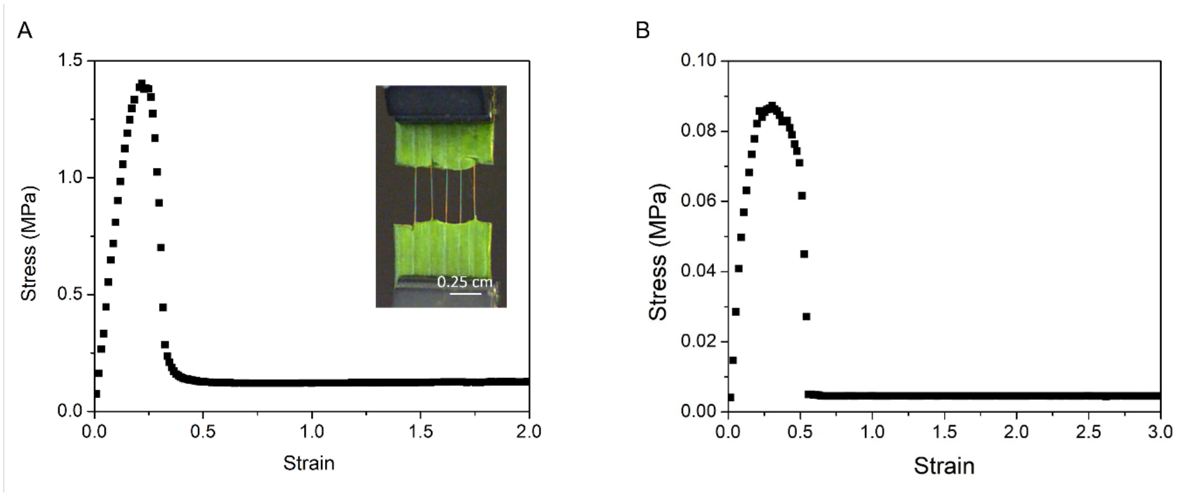

Fig. S4. *A. africanus* and *O. thyrsoides* leaves tensile essays. (*A* and *B*) Typical stress-strain uniaxial curves of *A. africanus and O. thyrsoides* leaves, respectively. Measurements made parallel to the direction of the treachery orientation at a strain rate of 5 mm/min. The leaves are brittle with a Young´s modulus of 8.1±0.1 MPa and a strain at break of 0.218±0.001, for *A. africanus* leaves and 0.41±0.01 MPa and a strain at break of 0.49±0.01, for *O. thyrsoides* leaves. The non-zero plateau observed for higher strains, after breaking the leaves, is due to the extraction of the treachery microfilaments and formation of bundles. The inset in *A* shows the two portions of a leaf between clamps with five microfilamentary bundles, after the tensile mechanical essay.

Fig. S5

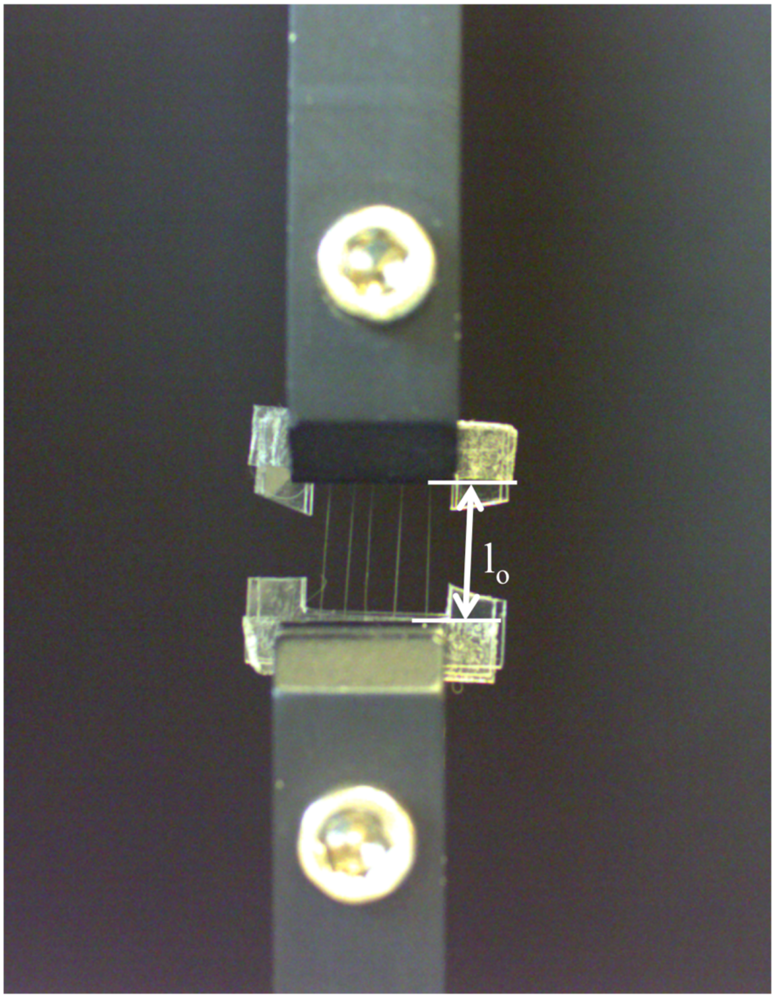

Fig. S5. Detail showing the bundles fixed between two clamps before the mechanical tensile tests. l_o=6.5 mm was their initial length.

Table S1

Table S1 AFM nano indention values of the elastic modulus and adhesion for isolated microfilaments extracted from *A. africanus* (A) and *O. thyrsoides* (O) leaves.

	Elastic modulus (MPa)		Adhesion (µN)	
	A	O	A	O
Average	0.74	0.50	0.27	0.02
Std. deviation	0.09	0.16	0.05	0.02
3rd Quartile	0.80	0.60	0.30	0.03
Median	0.76	0.50	0.20	0.02
1st Quartile	0.67	0.35	0.23	0.01
